# Atorvastatin lowers serum calcium levels in lithium-users: results from a randomized controlled trial

**DOI:** 10.1186/s12902-022-01145-w

**Published:** 2022-09-24

**Authors:** Jocelyn Fotso Soh, Katie Bodenstein, Oriana Hoi Yun Yu, Outi Linnaranta, Suzane Renaud, Artin Mahdanian, Chien-Lin Su, Istvan Mucsi, Benoit Mulsant, Nathan Herrmann, Tarek Rajji, Serge Beaulieu, Harmehr Sekhon, Soham Rej

**Affiliations:** 1 GeriPARTy Research Group, Jewish General Hospital, Montreal, Canada; 2 Department of Psychology, Concordia University, Montreal, Canada; 3 Department of Psychiatry, McGill University, 1033 Avenue des Pins, Montreal, H3A 1A1 Canada; 4 Centre for Clinical Epidemiology, Lady Davis Institute, Jewish General Hospital, Montreal, Quebec Canada; 5 Division of Endocrinology and Metabolism, Jewish General Hospital, McGill University, Montreal, Quebec Canada; 6 Douglas Mental Health University Institute, Montreal, Quebec Canada; 7 National Institute for Health and Welfare, Helsinki, Finland; 8 Department of Psychiatry, Jewish General Hospital, Montreal, Canada; 9 Epidemiology, Biostatistics and Occupational Health, McGill University, Montreal, Canada; 10 Multiorgan Transplant Program, University Health Network and Division of Nephrology, Department of Medicine, University of Toronto, Toronto, Canada; 11 Department of Psychiatry, University of Toronto, Toronto, Canada; 12 Department of Psychiatry, Centre for Addictions and Mental Health, University of Toronto, Toronto, Canada; 13 Department of Psychiatry, Sunnybrook Health Sciences Centre, University of Toronto, Toronto, Canada; 14 Postdoctoral Research Fellow, Department of Medicine, Faculty of Medicine, McGill University, Montreal, Quebec Canada; 15 McGill Meditation and Mind-Body Medicine Research Clinic (MMMM-RC), Montreal, Canada

**Keywords:** Hypercalcemia, Hyperparathyroidism, Atorvastatin, lithium use, Calcium, Bipolar disorder

## Abstract

**Background:**

Although lithium is considered the gold-standard treatment for bipolar disorder (BD), it is associated with a variety of major endocrine and metabolic side effects, including parathyroid hormone (PTH) dependent hypercalcemia. Aside from surgery and medication discontinuation, there are limited treatments for hypercalcemia. This paper will assess data from a randomized controlled trial (RCT).

**Methods:**

This is a secondary analysis of an RCT that explored the effects of atorvastatin (*n* = 27) versus placebo (*n* = 33) on lithium-induced nephrogenic diabetes insipidus (NDI) in patients with BD and major depressive disorder (MDD) using lithium (*n* = 60), over a 12-week period. This secondary analysis will explore serum calcium levels and thyroid stimulating hormone (TSH) measured at baseline, week 4, and week 12.

**Results:**

At 12-weeks follow-up while adjusting results for baseline, linear regression analyses found that corrected serum calcium levels were significantly lower in the treatment group (mean (M) = 2.30 mmol/L, standard deviation (SD) = 0.07) compared to the placebo group (M = 2.33 mmol/L, SD = 0.07) (β = − 0.03 (95% C.I.; − 0.0662, − 0.0035), *p* = 0.03) for lithium users. There were no significant changes in TSH.

**Conclusion:**

In lithium users with relatively normal calcium levels, receiving atorvastatin was associated with a decrease in serum calcium levels. Although exciting, this is a preliminary finding that needs further investigation with hypercalcemic patients. Future RCTs could examine whether atorvastatin can treat PTH dependent hypercalcemia due to lithium and other causes.

## Background

Bipolar disorder (BD) affects over 800,000 Canadians at least once in their life [1]. Lithium is considered the gold standard treatment for BD and treatment-resistant major depressive disorder (MDD), with 30–40% of patients responding better to lithium than any other treatments [2, 3]. However, lithium’s use has declined in the last three decades due to the risk of chronic kidney disease and other adverse effects, with only 8% of BD patients currently receiving this drug in America [4–7]. There are complementary and alternative therapies that can be used in conjunction with medications for BD, but lithium remains as the gold standard [8].

One of lithium’s important endocrine and metabolic adverse effects is hypercalcemia [9]. Health Canada and the U.S Food and Drug Administration (FDA) issued a black-box warning for lithium highlighting the risk of hypercalcemia and hyperparathyroidism [10, 11]. They have advised monitoring calcium levels throughout treatment and if hypercalcemia occurs, lithium withdrawal may be necessary as there are limited treatment options [11]. Approximately 2.0% of non-geriatric and 5.1% of geriatric lithium users have hypercalcemia and 23% of users have elevated PTH levels [12]. Lithium can alter calcium concentrations by increasing reabsorption of calcium within the loop of Henle and may also interfere with feedback mechanisms in the parathyroid, therefore preventing the suppression of parathyroid hormone (PTH) when calcium levels are high [13]. Hypercalcemia and hyperparathyroidism, can lead to acute kidney injury, chronic kidney disease, nervous system defects, and decreased skeletal health [4, 5, 14]. To date, surgery is the recommended treatment for hyperparathyroidism for patients with parathyroid adenomas and hyperplasia with symptoms caused by hypercalcemia [15]. The other unpalatable option to treat lithium-associated hypercalcemia is lithium discontinuation, which is associated with very high (> 33–50%) rates of mood disorder relapse [16, 17].

An additional metabolic side effect of lithium is hypothyroidism [4]. Lithium users have a two-fold increased risk of hypothyroidism in comparison to the general population [12], and 33% of chronic lithium users over 65 years of age developed hypothyroidism, usually within 1–7 years of initiation [18].

New treatments for lithium-associated adverse effects are needed. Observational and animal data suggest that statins may be helpful to improve lithium-induced nephrogenic diabetes insipidus (NDI) [19–21]. NDI is closely linked to other adverse effects associated with lithium use [22], and can have shared mechanisms (e.g. GSK3Beta inhibition with lithium use has been linked to effects on kidney function, diabetes, cancer) [23–25]. Thus, statins, given their pleiotropic effects, could be explored as a potential candidate for reducing serum calcium and other endocrine/metabolic adverse effects.

This secondary analysis of a randomized controlled trial (RCT) will assess whether atorvastatin use in lithium users can have an effect on serum calcium and thyroid stimulating hormone (TSH) [26–28].

## Methods

### Participants and study design

Data will be used from a pre-existing large double blind RCT conducted on lithium users to assess the effect of atorvastatin (20 mg/day) vs placebo on lithium induced NDI in patients with BD (*n* = 54) and MDD (*n* = 6). This double-blind RCT (Clinicaltrials.gov ID: NCT02967653) was conducted over a 12-week period [26–28]. For this secondary analysis, the RCT data will be used to assess whether atorvastatin versus placebo can reduce calcium levels in lithium users with BD and MDD. This study was approved by the local research ethics board at Douglas Mental Health University Institute (DMHUI), McGill University Health Centre (MUHC), and Jewish General Hospital (JGH) in Montreal, Canada and took place at these hospitals.

Participants were required to provide written informed consent prior to participating. Participants were considered eligible if they met the following inclusion criteria: 18–85 years old, psychiatric diagnosis of BD or MDD, currently using lithium for at least 2 months prior, and have partial NDI (urine osmolality of < 600 mOsm/Kg) or complete NDI (urine osmolality of < 300 mOsm/Kg). Participants not meeting this criteria were excluded from the study. A sample of *n* = 60 participants will allow an effect size of 0.34 at two-tailed alpha = 0.05 and Power (1-Beta) =0.8 [28]. For the detailed study methodology and Consort Diagram, refer to the study protocol and the original RCT [26–28].

### Intervention and control group

Participants were randomly assigned 1:1 to the intervention group (20 mg/day atorvastatin for 12 weeks) or placebo control group. The placebo control group received a placebo pill that was similar to the atorvastatin pill for the duration of the 12 weeks. Over the 12-week period, participants were closely monitored and required to visit the data collection site (DMHUI, JGH or MUHC) at baseline, week 4, and week 12 for testing.

### Outcomes

Upon each visit, blood and urine samples were collected after a mandatory 10-hour water restriction and a 12-hour fasting period. A variety of other tests took place to ensure safety of patients, including mood assessments and questionnaires (refer to study protocol and original RCT) [28].

This secondary analysis will assess serum calcium, thyroid stimulating hormone (TSH), and low-density lipoproteins (LDL) that were previously collected in the original RCT. LDL was previously reported in the original RCT [27]. Established literature and medical guidelines were used to determine which values were considered within normal ranges for all blood and urine tests [29–31].

The outcomes for this study are changes in serum calcium levels and TSH at 12-weeks follow-up adjusted for baseline in the atorvastatin compared to placebo group. LDL levels will be used as a proxy for atorvastatin treatment adherence (in addition to pill counts). All lab tests were collected at the same laboratory (DMHUI) throughout the study.

### Data analysis

Demographic characteristics were described using descriptive statistics (e.g., mean and standard deviation or frequency and percentages). To assess adequacy of randomization, baseline clinical and demographic characteristics were compared in the treatment versus placebo group using Chi-square, Mann-Whitney U, and t-tests. Normality was assessed using the Shapiro-Wilk Test and a linear regression model was used to assess the relationship between continuous variables. For the study outcomes, linear regressions were used to assess the differences in the treatment versus placebo group between the adjusted baseline and week 12. For this analysis, a linear regression model is used where the change in calcium from baseline to 12 week is treated as a dependent variable while adjusting for possible confounders/covariates and baseline value. The current international standard used by biostatisticians recommends that when assessing continuous variables in RCTs that a linear regression of raw scores adjusted for baselines should be used [32]. All statistical analyses were computed on The R Project and Statistical Package for the Social Science (SPSS) software version 25.0. Statistical significance was set to a *p*-value of less than or equal to 0.05.

## Results

### Demographic characteristics

A total of 60 participants (35 females, 25 males) were recruited for this study, with four participants dropping out (Retention Rate = 93.3%). There were 27 participants (48.1% females, 51.9% males) who were randomly assigned to the treatment group and 33 (66.7% females, 33.3% males) assigned to the placebo group. There were no significant differences in demographics characteristics between the treatment and placebo group except that participants in the treatment group had: a higher degree of education (χ^2^ = 9.696, *p* = 0.021) and a lower prevalence of antidepressant use (χ^2^ = 4.42, *p* = 0.035). Table 1 shows additional demographic and clinical characteristics of the study.

**Table 1:** Mean and standard deviation values or percent and number of participants for demographic characteristics in the treatment and placebo group

Variable	Atorvastatin Group (*n* = 27) Mean (SD) or %(n)	Placebo Group (*n* = 33) Mean (SD) or %(n)
**Demographic Characteristics**		
Mean Age	47.81 (13.75)	53.12 (11.79)
Gender		
Female	48.1% (*n* = 13)	66.7% (*n* = 22)
Male	51.9% (*n* = 14)	33.3% (*n* = 11)
Level of Education		
Elementary School	1.6% (*n* = 1)	0% (*n* = 0)
High School	16.7% (*n* = 10)	26.7% (*n* = 16)
University (Bachelor)	16.7% (*n* = 10)	28.3% (*n* = 17)
University (Professional, Graduate Degree)	10.0% (*n* = 6)	0% (*n* = 0)
**Lithium Use Variables**		
Lithium Dose (First visit)	857.69 (246.85)	761.66 (323.95)
Serum Lithium Level (First visit)	0.607 (0.16)	0.603 (0.19)
**Psychiatric and Medical History**		
Bipolar Disorder	88.88% (*n* = 24)	90.9% (*n* = 30)
Unipolar Depression	11.11% (*n* = 3)	9.09% (*n* = 3)
Number of past mood episodes	4.92 (1.62)	5.0 (1.52)
Number of past manic episodes	3.11 (2.26)	4.12 (3.35)
Number of Medications	4.89 (1.50)	4.82 (1.45)
Number of psychotropics medications	3.52 (1.87)	3.64 (1.75)
Patients taking antipsychotics	0.70 (0.47)	0.67 (0.48)
Patients taking antidepressants	0.33 (0.48)	0.61 (0.50)
Patients taking Valproate	0.15 (0.36)	0.06 (0.24)
Patients taking Lamotrigine	0.19 (0.40)	0.24 (0.44)
Patients taking Benzodiazepines	0.15 (0.36)	0.24 (0.44)

### Study outcome

**Fig. 1:**
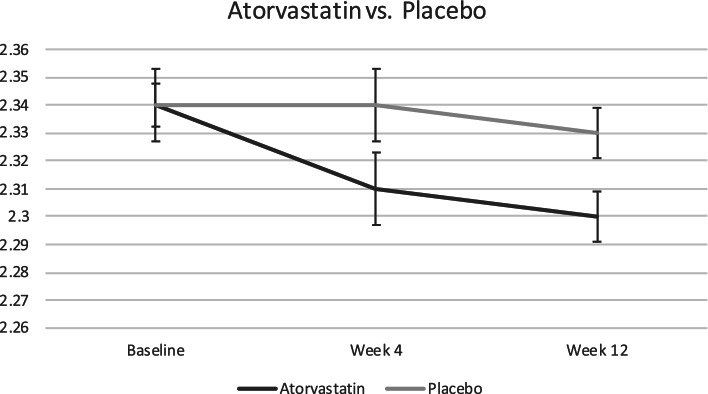
Change in serum calcium (mmol/L) in Atorvastatin versus placebo at baseline, week 4, and week 12 with standard error bars

After adjusting for baseline values, corrected calcium levels at 12-week follow-up were significantly lower in the treatment group (Mean (M) = 2.30 mmol/L, Standard Deviations (SD) = 0.07) compared to the placebo group (M = 2.33 mmol/L, SD = 0.07) (β = − 0.03 (95% C.I.; − 0.07, − 0.0035), *p* = 0.03) (Table 2, Fig. 1). There were no reports of patients with hypercalcemic or hypocalcemic serum calcium levels in this sample at baseline – the maximum serum calcium level was 2.62, with a minority of patients with a baseline serum calcium level *≥* 2.5 mmol/L, *n* = 2.

**Table 2:** Mean, standard deviation, confidence intervals, and *p*-values for the study outcomes for the treatment and placebo group

Variable	Visit	Atorvastatin Mean (SD)	Placebo Mean (SD)	Statistics ${\hat{\beta}}_1$ (95% CI)
Corrected Serum Calcium (mmol/L)	1	2.34 (0.06)	2.34 (0.10)	−0.03 (−0.0662, − 0.0035) ***p*** **= 0.0336**
	2	2.31 (0.10)	2.34 (0.10)	
	3	2.30 (0.07)	2.33 (0.07)	
TSH (mU/L)	1	2.12 (1.27)	2.36 (1.19)	−0.27 (−0.8467, 0.3029) *p* = 0.3582
	2	5.86 (18.70)	2.30 (1.29)	
	3	1.89 (1.19)	2.34 (1.23)	
LDL (mmol/L)	1	2.60 (0.67)	2.98 (0.99)	−1.07 (−1.3793, −0.7661) ***p*** **= 4.85 × 10**^**−9**^
	2	1.76 (0.60)	3.05 (1.01)	
	3	1.69 (0.79)	3.07 (0.98)

No statistical significance was found between the treatment and placebo group for TSH (*p* > 0.05) (Table 2).

As expected, LDL was significantly reduced in the atorvastatin treatment group (M = 1.69 mmol/L, SD = 0.79) compared to placebo (M = 3.07 mmol/L, SD = 0.98) at week 12 (β = − 1.07 (95% C.I.; − 1.38, − 0.77), *p* = 4.85 × 10^− 9^).

## Discussion

This is the first randomized controlled trial, to our knowledge, that has demonstrated the potential of atorvastatin to lower serum calcium levels in lithium users. In fact, we are not aware of any human or animal studies that have evaluated the effect of statins on serum calcium levels. The main finding of our study was that serum calcium levels had significantly decreased in individuals using atorvastatin compared to individuals in the placebo group who were using lithium. These results provide helpful preliminary information that can lead to future large RCTs with hypercalcemic patients.

The mechanism by which atorvastatin could improve calcium levels remains unclear. The ability of statins to have pleiotropic mechanisms such as with cancer, testosterone, androgens, diabetes, and more, makes this a unique drug [33–35]. It appears that several lithium-associated adverse effects, including metabolic ones, occur concurrently with NDI [22]. This suggests a similar biological mechanism to NDI, such as effects on GSK3β or intracellular calcium signalling pathways. Lithium decreases the aquaporin 2 protein, leading to NDI, which can also affect calcium levels. Statins can reverse this effect by increasing aquaporin 2, which may play a role in affecting calcium levels [36]. The exact biological mechanisms could be explored further.

Since we processed all calcium and other lab tests at a single laboratory (DMHUI) and the standard deviations were very narrow, laboratory error is unlikely. However, a mean decrease of 0.03 mmol/L relative to placebo may be of limited clinical relevance. Considering this study had a relatively normo-calcemic sample of lithium users, it is possible that atorvastatin’s potential effects on serum calcium were less prominent. Future RCTs can investigate the effect of atorvastatin on participants with diagnosed hypercalcemia. The inclusion/exclusion criteria required participants to be taking lithium for at least two months prior to the start of the study, however the participants were mostly long-term lithium users with more than 5–10 years of lithium use (average age 50.7 years). Lithium adverse effects are generally more reversible when lithium use is < 5–10 years [37], therefore these effects of lowering serum calcium levels may be more prominent in other samples with patients who have more recently initiated lithium. To assess clinical significance, studies must assess the effect of statins on patients with hypercalcemia or recent initiation of lithium.

There were no significant effects of atorvastatin on TSH. According to the literature, statins could decrease TSH levels [38], or could have no significant effect on TSH levels [39]. Hypothyroidism has been described to occur during the first seven years of lithium treatment and it is often treated by lithium discontinuation or thyroid replacement therapy once hypothyroidism is diagnosed [18, 40]. The effect of statins may have been less compelling in our short-term treatment trial but could be tested in a longer-term lithium-associated hypothyroidism prevention study. Future RCTs can also monitor triiodothyronine (T3) and thyroxine (T4) to examine the mechanism by which atorvastatin affects the thyroid.

Statins are traditionally used to reduce cholesterol levels, specifically LDL. As the levels of LDL were significantly reduced in the treatment group after receiving atorvastatin, relative to the placebo group, it indicated that there was patient adherence in taking atorvastatin.

### Strengths and limitations

A strength of this study was the use of a randomized double-blind placebo-controlled study design. This type of design allowed for the mitigation of potential biases in researchers and participants (e.g., demand characteristics). Additionally, the placebo-controlled and longitudinal aspect of this study was useful for baseline comparisons to measure the actual effect of the treatment group over time. One possible limitation is that this sample only included participants with partial or complete NDI (the parent RCT was designed to assess whether atorvastatin could treat NDI), which may theoretically limit generalizability to non-NDI lithium users. Another limitation was the modest sample size and small effect size. Since this was a secondary analysis of a parent RCT, we were unfortunately missing systematic data examining vitamin D, dietary calcium intake, food frequency questionnaire, 24-hour dietary recall, 3-day dietary record, serum PTH, serum proteins, serum albumin, urinary calcium, ACE inhibitors/ARBs, loop diuretics or NSAIDs [41, 42]. Our findings that serum calcium statistically decreased in atorvastatin users were nonetheless exciting, and the next steps would be examining the effect of atorvastatin while measuring for these variables in a sample with hyperparathyroidism and hypercalcemia. Hyperparathyroidism is relatively more common in older adults and females – our findings may be particularly relevant in that subpopulation of lithium users, which could be assessed in future studies.

## Conclusion

We found that atorvastatin use may reduce serum calcium levels in lithium users. This study was the first to demonstrate a link between statin effects and calcium. This effect occurred rapidly within 12 weeks of treatment on a normo-calcemic sample, thus, future studies are needed to assess proper dosing and duration of atorvastatin use in a hypercalcemic sample. We propose that the calcium-reducing effects of statins may not solely be limited to lithium users and may be beneficial to other forms of hyperparathyroidism and PTH-dependent hypercalcemia. Future studies can assess clinical significance, explore mechanism behind statin pathway and calcium metabolism, and confirm whether statins can reduce calcium in patients with hypercalcemia.

## Data Availability

Restrictions apply to some or all the availability of data generated or analyzed during this study to preserve patient confidentiality or because they were used under license. The corresponding author will on request detail the restrictions and any conditions under which access to some data may be provided.

## Abbreviations

BD: Bipolar disorder
PTH: Parathyroid hormone
RCT: Randomized controlled trial
TSH: Thyroid stimulating hormone
M: Mean
SD: Standard deviation
FDA: Food and Drug Administration
HbA1c: Hemoglobin A1C
NDI: Nephrogenic diabetes insipidus
DMHUI: Douglas Mental Health University Institute
JGH: Jewish General Hospital
MUHC: McGill University Health Centre
LDL: low-density lipoproteins
BMI: Body mass index
SPSS: Statistical Package for the Social Science
T3: Triiodothyronine
T4: Thyroxine
